# Rasch Analysis of Self-Reported Adherence to Patient-Centered Physical Therapy Scale among Japanese Physical Therapists: Cross-Sectional Study

**DOI:** 10.3390/ijerph181910282

**Published:** 2021-09-29

**Authors:** Hiroshi Takasaki

**Affiliations:** Department of Physical Therapy, Saitama Prefectural University, Koshigaya 343-8540, Japan; physical.therapy.takasaki@gmail.com; Tel.: +81-489734706

**Keywords:** adherence, patient-centered approach, physical therapy, Rasch analysis, unidimensionality

## Abstract

This study primarily aimed to develop a shorter version of the self-reported adherence to patient-centered physical therapy (s-SAPCPTS) by using Rasch analysis and secondarily aimed to preliminarily investigate the relationship between the s-SAPCPTS scores and demographics (i.e., age, sex, final academic degree (non-postgraduate degrees or postgraduate degrees), and practice environment). In an online anonymous survey, 110 Japanese physical therapists completed the self-reported adherence to patient-centered physical therapy and provided data on their demographics. Through the Rasch analysis, items were excluded in a stepwise manner, until certain pre-established criteria of the unidimensionality were satisfied. Subsequently, a conversion table for the Rasch score was developed. Furthermore, multiple regression analysis was conducted by using the independent variables age, sex, and final academic degree. Using the Kruskal–Wallis test, we compared the Rasch s-SAPCPTS scores among four practice environments. Consequently, the seven-item s-SAPCPTS was developed by excluding seven items through the Rasch analysis. Postgraduate degree was a statistically significant contributing factor for Rasch s-SAPCPTS scores (*p* = 0.038, *β* = 0.20). The Kruskal–Wallis test demonstrated statistically significant differences in the Rasch s-SAPCPTS scores among the four practice environments (*p* = 0.006). In conclusion, the seven-item s-SAPCPTS was developed with the preliminary evidence of construct validity. It was also found that the final academic degree and practice environment could be the contributing factors of s-SAPCPTS scores.

## 1. Introduction

Person-centered approaches, where persons (patients) actively participate in their health service, increase patient satisfaction and exercise adherence [1,2,3,4]. The importance of person-centered approaches has been recognized among guideline developers [5] and included in one of six core concepts to improve a health care system [6]. However, in physical therapy practice, person-centered approaches have not always been provided, due to their preference for a biomedical approach that limits the person-centered approach [7,8,9]. One of the initial steps in the facilitation of a person-centered approach in physical therapy is to develop a simple self-reporting measure for adherence to person-centered approaches to allow us to investigate the effect of educational interventions.

In 2019, Shand, et al. [10] developed the Healthcare Providers Patient-Activation Scale (HP-PAS) to evaluate attitudes toward patient-activation; the items in the scale were generated from ecological perspectives of patient self-management proposed by Fisher et al. [11]. In the HP-PAS, 20 items relevant to person-centered approaches were selected to evaluate the importance toward the person-centered approach. Subsequently, it was found that 14 out of the 20 items in the Japanese version of the HP-PAS [12] demonstrated content validity with adequate test–retest reliability, when the response scale was changed from a five-point Likert scale for the importance to an 11-point numerical rating scale (NRS) (i.e., 0–100%) for the adherence [13]. Consequently, the 14-item questionnaire was proposed to evaluate the self-reported adherence to patient-centered physical therapy (SAPCPTS). However, evaluation of the use of the 11-point NRS is required as Simms, et al. [14] suggested that the choice of response scale was important and there was no advantage for any response scales beyond six options. Further, the construct validity of the SAPCPTS has not been examined till date. To allow therapists to calculate sum scores for comparing the magnitude of the SAPCPTS, the unidimensionality of the scale needs to be investigated. The appropriateness of the scale and the unidimensionality can be assessed by using the Rasch analysis.

Furthermore, it was considered important to investigate whether the demographics correlate with the magnitude of the SAPCPTS to consider future strategies of facilitating PCA in physical therapy. Apart from the basic demographics including age and sex, the final academic degree was suspected to be a relevant factor, as final academic degrees influence adherence to the clinical practice guideline [15]. The practice environment was also suspected as a relevant factor for the SAPCPTS, because the working environment can be a relevant factor for evidence-based practice [16,17].

The primary aim of the current study was to develop a shorter version of the SAPCPTS (s-SAPCPTS) by assessing appropriateness of the response scale and unidimensionality. The secondary aim was to investigate the relationship between s-SAPCPTS scores and demographics.

## 2. Materials and Methods

### 2.1. Design

The s-SAPCPTS was developed by reducing items that affect unidimensionality, using the Rasch analysis. Furthermore, test–retest reliability of the total score of s-SAPCPTS and its minimum detectable changes (MDCs) were calculated by using shared datasets of 53 participants from a previous study [13] who were recruited by using the same inclusion criteria as those in the current study. This study was approved by the institutional research ethics committee (Saitama Prefectural University; protocol code: #20011).

### 2.2. Participants

Data were collected between July and September 2020 via an anonymous online survey posted on the author’s personal webpage (https://physicaltherapytak.wixsite.com/mysite, accessed on 30 August 2021). An online link to the survey was posted on Facebook. The inclusion criteria were (1) possession of Japanese physical therapist credentials and (2) knowledge of Japanese as the native language.

### 2.3. Outcomes

The primary outcome was the 14-item SAPCPTS (Supplementary Materials Table S1). Respondents rated self-reporting adherence to each item in their clinical practice in percentage, using an 11-point NRS from 0% (never) to 100% (always) with 10% intervals. Higher total scores indicated greater adherence to patient-centered physical therapy.

The secondary outcomes were demographics, including age, sex, final academic degree (non-postgraduate degrees including diploma and bachelor degrees or postgraduate degrees including master’s and doctorate degrees), practice environment (hospital; clinic; long-term care health facilities, nursing home, or others; or educational institute).

### 2.4. Procedures

Data collection was continued until a minimum sample of 100 was obtained, which is considered acceptable to run Rasch analysis [18], adequate to construct validity in the Consensus-Based Standards for the Selection of Health Measurement Instruments [19,20], and acceptable to perform multiple regression analysis with three dependent variables (i.e., *n* = 15–30 per dependent variable [21]).

In the 14-item SAPCPTS, Rasch analysis was conducted by using the Andrich’s Rating Scale Model with the Winsteps version 3.93 (Winsteps.com, Beaverton, Oregon). Unidimensionality was assessed by using the criteria reported in previous studies [22,23,24,25]. Briefly, the response format was considered appropriate when (1) all response options had >10 counts, (2) average measures of person abilities increased with response options, (3) outfit mean square (MnSq) values of each response option were <2, and (4) there was no disordering step calibration [22,24]. The response options were modified when the criteria were not satisfied. Subsequently, unidimensionality was considered when all following criteria were satisfied: (1) the eigenvalue was <2 in the first contrast, and (3) infit/outfit MnSq statistics was <1.4 and standard Z-values were <2. An item with a MnSq of >1.4 and a standard Z-value of >2 indicated a construct different from other items and thus was excluded in a stepwise manner until the criteria of unidimensionality were satisfied. Consequently, the s-SAPCPTS was developed.

The response distribution of the s-SAPCPTS was also assessed by visualizing an item–person map and assessing floor and ceiling effects. A threshold of 15% was used for the assessment of floor and ceiling effects [22,23]. Furthermore, the Rasch score of 0–100 was established.

Internal consistency, test–retest reliability, multiple regression analysis, and comparison among practice environments were assessed by using SPSS version 21.0 (IBM Corp, Armonk, New York), with a statistical significance of 5%. Internal consistency was assessed with Cronbach’s *α*, where *α* > 0.7 was considered acceptable [26]. Regarding test–retest reliability, the total Rasch s-SAPCPTS scores that were extracted from the datasets of 53 participants in a previous study [13] were used to obtain intra-class correlation coefficients (ICC), where the criteria for ICC value were as follows: ≤0.40 = weak, 0.41–0.74 = moderate, and ≥0.75 = strong [26]. Subsequently, the MDC in the Rasch s-SAPCPTS scores were calculated by using the following formulas:

(1) SD=standard deviations of 110 participants in the current study

(2) $$MDC=SD\sqrt{1-ICC}\times 1.96\times \sqrt{2}$$

To investigate relationships between the demographics of age, sex, and final academic degree and the Rasch s-SAPCPTS scores, multiple regression analysis was conducted by using the enter method. For data on sex and final academic degree, a 0/1 dummy code was used. For comparing the four practice environments, the Kruskal–Wallis test was performed by using the Rasch s-SAPCPTS scores, considering the uncertainty of normal distribution in each practice environment.

## 3. Results

In total, 110 participants completed the survey. Demographics of all patients are summarized in Table 1. The criteria were satisfied with the 11-point NRS in the seven-item s-SAPCPTS. To satisfy the criteria of unidimensionality, seven items were excluded, and consequently, a seven-item s-SAPCPTS was developed. The items in the original English version of s-SAPCPTS, as well as its Japanese version, are presented in Supplementary Materials Table S2. In the s-SAPCPTS, the eigenvalue of the first contrast was 1.78, and 66.2% of the raw variance was explained by the measure. Table 2 presents fit statistics in the s-SAPCPTS.

**Figure 1:**
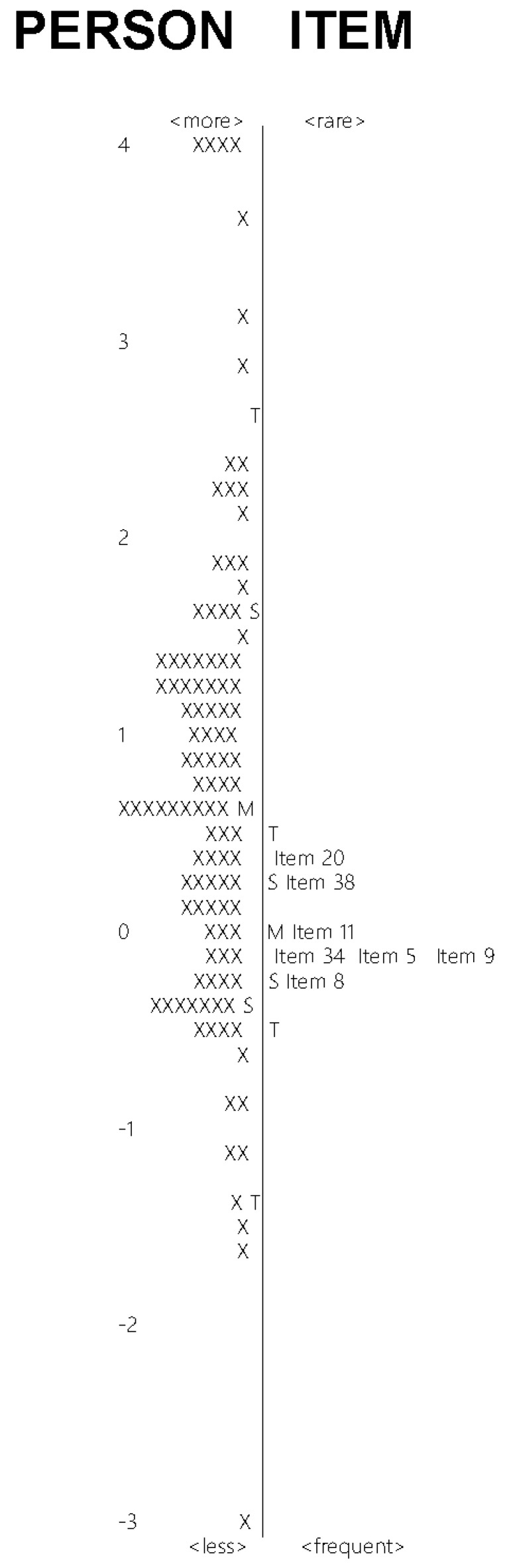
Rasch item–person map.

Neither ceiling (5.7%) nor flooring effects (1.4%) were observed. Figure 1 demonstrates the Rasch item–person map. The mean of person ability appeared close to the mean of item difficulty; however, the distribution of item difficulty did not cover that of person ability. The conversion from the raw total score to the 0–100 Rasch score of the s-SAPCPTS is presented in Supplementary Materials Table S3.

The Cronbach-*α* was 0.93, indicating acceptable internal consistency. The ICC (95 confidence interval) of the s-SAPCPTS was 0.82 (0.71–0.89) and the MDC was 17.21.

As demonstrated by the multiple regression modeling, the final academic degree was a statistically significant contributing factor for Rasch s-SAPCPTS scores (Table 3). There were two outliers for which the predicted value of the measured value was above ±3 standard deviations.

**Figure 2:**
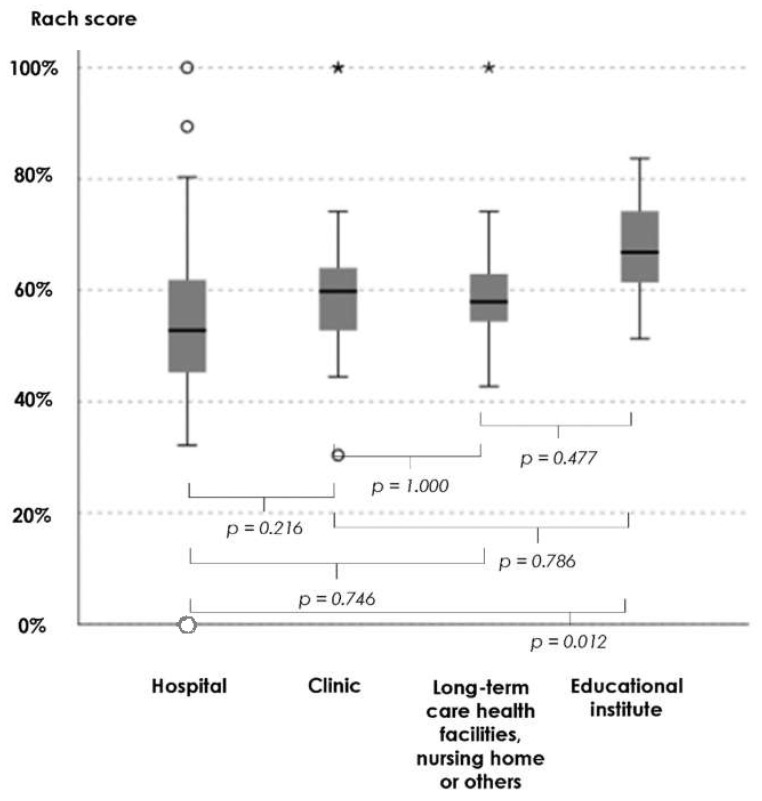
Kruskal–Wallis test for the Rasch score of the seven-item Self-Reported Adherence to Patient-Centered Physical Therapy Scale among the four practice environments. ◯ outliers and * extreme outliers.

The Kruskal–Wallis test demonstrated statistically significant differences in the Rasch s-SAPCPTS scores among the four practice environments (*p* = 0.006). Pairwise multiple comparison test with Bonferroni correction demonstrated a statistically significant difference only between the practice environments of hospital and educational institute (*p* = 0.012) (Figure 2).

## 4. Discussion

In the current study, we developed the s-SAPCPTS for Japanese physical therapists via the confirmation of appropriateness of the 11-point NRS and unidimensionality. The s-SAPCPTS also demonstrated acceptable internal consistency, test–retest reliability, and neither ceiling nor flooring effect. The Rasch item–person map showed relatively matched mean person ability and item difficulty, and limited distribution of item difficulty. These characteristics are not surprising, because the included items were limited to only seven. Although it is optimal that the distribution of item difficulty completely covers the distribution of person ability, it would be difficult to deny the construct validity of the s-SAPCPTS by using the biased item–person map only, considering the relatively matched mean person ability and item difficulty. Thus, the current study demonstrated preliminary evidence of validity and reliability of the s-SAPCPTS and suggests clinical use of the scale in the future.

Interestingly, neither age nor sex, but instead, the final academic degree and practice environment were the contributing factors for the Rasch s-SAPCPTS score. Physical therapists in the education institute had the highest Rasch s-SAPCPTS score, which is not surprising, considering that physical therapists in the education institute often have postgraduate degrees. These findings correspond to those of a previous study conducted among Japanese physical therapists [15], in which postgraduate education in the Mechanical Diagnosis and Therapy (MDT) was a factor contributing to biopsychosocially oriented approaches. Similarly, among Japanese physical therapists, clinical experience and sex were not the contributing factors for identifying the psychological status of the patient through physical evaluation without a questionnaire [27], which was possible in therapists with the highest MDT training [28]. Thus, in Japanese physical therapists, post-graduate training is considered useful for increasing s-SAPCPTS scores, which may result in the implementation of person-centered approaches.

### 4.1. Research Agenda

This study found that the final academic degree was a statistically significant contributing factor for s-SAPCPTS scores, but the effect size of *R*^2^ = 0.09 can be interpreted as a none-to-very weak effect size [29]. These findings indicate that other factors that are relevant to the final academic degree may better influence the s-SAPCPTS scores, for example, pain neurophysiology knowledge [30,31] and adherence to evidence-based practice [16,32]. Further, skills for behavioral modifications, which can be enhanced in post-graduate clinical training, for example, skills to enhance patient’s attitude toward self-management [33] and communication skills to enhance patient autonomy [34], may better influence the s-SAPCPTS scores than the academic degree. Further studies are required to identify important factors to facilitate the implementation of person-centered approaches.

### 4.2. Limitations

A limitation of the current study is the generalizability of the scale. Educational levels influence the magnitude of the self-reported adherence of the person-centered approach; thus, s-SAPCTPRS scores of Japanese physical therapists could be different from those of physical therapists in other countries, considering differences in database use [35]. Another limitation is a potential bias in sampling. The data used in this study were not collected by all physical therapists in a certain community, such as the Japanese Physical Therapy Association; thus, there could have been self-selection bias and self-presentation bias. Furthermore, the effect size in the multiple regression analysis can be interpreted as a none-to-very weak effect size [29]. Although robust contributing factors should be determined by using a more comprehensive sampling method with a far larger sample size and other promising dependent variables, the findings in the current study will be a foundation for future studies.

## 5. Conclusions

In this study, the seven-item s-SAPCPTS was developed by using preliminary evidence of construct validity. It was also found that the final academic degree and practice environment could be the contributing factors of s-SAPCPTS scores. The developed s-SAPCPTS has possible applications among Japanese physical therapists in future studies.

## Figures and Tables

**Table 1:** Demographics of the participants.

Variables	Rasch Analysis (*n* = 110)	Regression Modeling (*n* = 85)
Age (years), mean ± SD	32.5 ± 7.2	32.7 ± 7.1
Sex (no. of males to no. of females)	85:25	68:17
Final academic degree (*n*), [%]		
Doctorate	3 [2.7]	3 [3.5]
Master’s	17 [15.5]	15 [17.6]
Bachelor	53 [48.2]	38 [44.7]
Diploma	37 [33.6]	29 [34.1]
Years since the acquisition of the physical therapy license (years), mean ± SD	9.4 ± 6.0	9.8 ± 6.0

**Table 2:** Fit statistics in the seven-item Self-Reported Adherence to Patient-Centered Physical Therapy Scale.

Item No.^1^	Measure	SE	Infit MnSq	Infit Zstd	Outfit MsSq	Outfit Zstd
Item 20	0.42	0.07	1.03	0.3	1.05	0.4
Item 38	0.24	0.07	1.14	1.0	1.08	0.6
Item 11	0.01	0.07	1.04	0.4	1.05	0.4
Item 9	−0.07	0.07	0.90	−0.7	0.87	−0.9
Item 5	−0.15	0.07	0.82	−1.3	0.86	−1.0
Item 34	−0.15	0.07	1.05	0.4	0.96	−0.2
Item 8	−0.30	0.07	1.07	0.5	0.95	−0.3

^1^ Correspond with the 40-item Healthcare Providers Patient-Activation Scale [10]. Abbreviations: SE, standard error of measurement; MnSq, mean square; Zstd, standardized Z value.

**Table 3:** Results of multiple regression modeling for the Rasch score of the seven-item Self-Reported Adherence to Patient-Centered Physical Therapy Scale.

Model	Unstandardized Coefficients (*B*) (95% Confidence Intervals)	Standardized Coefficients (*β*)	*p*-Value
(Constant)	50.63 (36.14–65.12)		<0.001
Sex ^1^	−5.16 (−11.72–1.42)	−0.15	0.123
Final academic degree ^2^	7.58 (0.43–14.74)	0.20	0.038
Age	0.28 (−0.11–0.67)	0.14	0.154

*R*^2^ = 0.09, ANOVA *p* = 0.017, Durbin–Watson = 1.91; ^1^ 0 = female, and 1 = male; ^2^ 0 = non-postgraduate degrees, including diploma and bachelor degrees, and 1 = postgraduate degrees, including master’s and doctorate degrees.

## Data Availability

The data presented in this study are available on request from the corresponding author.

## Supplementary Materials

The following are available online at https://www.mdpi.com/article/10.3390/ijerph181910282/s1. Table S1: Descriptions of the 14-item Self-Reported Adherence to Patient-Centered Physical Therapy Scale. Table S2: Seven-item Self-Reported Adherence to Patient-Centered Physical Therapy Scale. Table S3: Conversion table from raw total scores to 0–100 Rasch scores.
