# Supplementary material for: Rasch Analysis of Self-Reported Adherence to Patient-Centered Physical Therapy Scale among Japanese Physical Therapists: Cross-Sectional Study
Source: Int J Environ Res Public Health. 2021 Sep 29;18(19):10282. doi: 10.3390/ijerph181910282 (PMC8508273; doi:10.3390/ijerph181910282)
Supplement: Supplementary file 1 [file ijerph-18-10282-s001.zip › Supplementary table S2.pdf]

Table S2. The seven-item Self-reported Adherence to Patient-Centered Physical Therapy Scale. Please remember your current clinical practice. Please mark the most suitable frequency associated with each tested item.

	0% (never)	10%	20%	30%	40%	50%	60%	70%	80%	90%	100% (always)
Support the patient to make decisions about what treatment options are best for them (Item 5)											
Help the patient to manage any impacts of their disease on their work or personal life (Item 8)											
Support the patient in identifying lifestyle changes they are willing to make (Item 9)											
Identify with the patient the skills they have to manage their symptoms (Item 11)											
Help the patient monitor their progress in making lifestyle change (Item 20)											
Help the patient identify what works for them to change their lifestyle (Item 34)											
Explore with the patient strategies for changing behavior (Item 38)											

Item numbers in the bracket correspond with the 40-item Healthcare Providers Patient-Activation Scale [1].

あなたの現在の臨床実践についてお聞きします。各文の内容を行なっている頻度について最も適当なところに印をつけてください。.

	0% (never)	10%	20%	30%	40%	50%	60%	70%	80%	90%	100% (always)
どのような治療選択が最適か患者が意思決定するのを支援する											
病気による仕事や私生活へのあらゆる影響に対して患者が対処できるように手助けをする											
患者が自ら変えようとしているライフスタイルは何か見出すのを手伝う											

自身の症状を管理するために、患者がどのようなスキルを有しているかを患者と一緒に確認する	
患者が自身のライフスタイル変更の進捗度を把握できるように手助けする	
ライフスタイルを変更するために何が効果的であるかを患者自身がわかるように手助けをする	
行動・ふるまいを変えるための方法を患者と一緒に探る	

Reference

1. Shand, L.; Higgins, R.; Murphy, B.; Jackson, A. Development and validation of the healthcare providers patient-activation scale. Patient Educ. Couns. 2019, 102, 1550–1557, doi:10.1016/j.pec.2019.03.005.
