# Supplementary material for: Rasch Analysis of Self-Reported Adherence to Patient-Centered Physical Therapy Scale among Japanese Physical Therapists: Cross-Sectional Study
Source: Int J Environ Res Public Health. 2021 Sep 29;18(19):10282. doi: 10.3390/ijerph181910282 (PMC8508273; doi:10.3390/ijerph181910282)
Supplement: Supplementary file 1 [file ijerph-18-10282-s001.zip › Supplementary table S3.pdf]

Table S3. Conversion table from raw total scores to 0-100 Rasch scores.

Raw total score	0-100 Rasch score	Raw total score	0-100 Rasch score	Raw total score	0-100 Rasch score
0	0	24	40.53	48	56.07
1	11.79	25	41.09	49	56.96
2	17.92	26	41.64	50	57.89
3	21.31	27	42.19	51	58.84
4	23.66	28	42.74	52	59.82
5	25.48	29	43.30	53	60.84
6	26.97	30	43.85	54	61.87
7	28.25	31	44.42	55	62.93
8	29.38	32	44.98	56	64.02
9	30.40	33	45.56	57	65.13
10	31.32	34	46.14	58	66.26
11	32.18	35	46.74	59	67.43
12	32.98	36	47.34	60	68.63
13	33.74	37	47.96	61	69.88
14	34.46	38	48.59	62	71.20
15	35.14	39	49.24	63	72.60
16	35.81	40	49.91	64	74.14
17	36.44	41	50.59	65	75.86
18	37.06	42	51.30	66	77.85
19	37.67	43	52.03	67	80.32
20	38.26	44	52.78	68	83.68
21	38.84	45	53.56	69	89.40
22	39.41	46	54.37	70	100
23	39.97	47	55.20		
