# Supplementary material for: Rasch Analysis of Self-Reported Adherence to Patient-Centered Physical Therapy Scale among Japanese Physical Therapists: Cross-Sectional Study
Source: Int J Environ Res Public Health. 2021 Sep 29;18(19):10282. doi: 10.3390/ijerph181910282 (PMC8508273; doi:10.3390/ijerph181910282)
Supplement: Supplementary file 1 [file ijerph-18-10282-s001.zip › Supplementary table S1.pdf]

Table S1. Descriptions of the 14-item Self-reported Adherence to Patient-Centered Physical Therapy Scale.

Item 4: Identify with the patient the barriers they are likely to face in making lifestyle change
Item 5: Support the patient to make decisions about what treatment options are best for them
Item 8: Help the patient to manage any impacts of their disease on their work or personal life
Item 9: Support the patient in identifying lifestyle changes they are willing to make
Item 11: Identify with the patient the skills they have to manage their symptoms
Item 17: Explore with the patient strategies for managing their emotions
Item 19: Develop a care plan with the patient for long-term management of their condition
Item 20: Help the patient monitor their progress in making lifestyle change
Item 29: Encourage the patient to seek information about managing their condition
Item 30: Identify with the patient their confidence to about managing their condition
Item 33: Explore with the patient strategies that have helped them manage their chronic condition in the past
Item 34: Help the patient identify what works for them to change their lifestyle
Item 35: Help the patient to monitor symptoms of their disease
Item 38: Explore with the patient strategies for changing behavior

Item numbers correspond with the 40-item Healthcare Providers Patient-Activation Scale [1].

Reference

1. Shand, L.; Higgins, R.; Murphy, B.; Jackson, A. Development and validation of the healthcare providers patient-activation scale. Patient Educ. Couns. 2019, 102, 1550–1557, doi:10.1016/j.pec.2019.03.005.
